# The Impact of Promotora Community Education and Text Messaging on Lowering Exposure to Environmental Toxins to Reduce Breast Cancer Risks and Reproductive Harm

**DOI:** 10.7759/cureus.94728

**Published:** 2025-10-16

**Authors:** Luisa R Blanco, Ashley Ramirez, Bianca Ojeda, Rebecca Martinez, Julie A Friedman, Estefania Ramires-Sanchez, Alexandra Klomhaus, Sophie A Viray, Gabrielle A Pascua, Janet Pregler

**Affiliations:** 1 School of Public Policy, Pepperdine University, Malibu, USA; 2 Department of Pediatrics, University of California Los Angeles, Los Angeles, USA; 3 Iris Cantor-UCLA Women's Health Education and Research Center, University of California Los Angeles, Los Angeles, USA; 4 Division of General Internal Medicine and Health Services Research, University of California Los Angeles, Los Angeles, USA

**Keywords:** environmental agents, environmental pollution, low-income settings, public health education, spanish-speaking, toxin

## Abstract

Introduction

Promotoras, lay health educators, are effective facilitators in improving health outcomes for Latinas in under-resourced communities. The Iris Cantor-UCLA Women’s Health Education and Research Center, in collaboration with Worksite Wellness Los Angeles, a non-profit organization, developed a training program in which Promotoras educated their family, friends, and community members on lowering the risk of breast cancer and reproductive harm by reducing exposures to toxins. Promotoras participated in four 90-minute weekly Zoom training sessions focused on the hidden hazards of environmental toxins related to reproductive health and breast cancer. Participants educated by Promotoras attended two educational sessions. The study aimed to determine if augmenting the educational training conducted by Promotoras with a digital text messaging component for the participants assigned to an intervention group resulted in higher knowledge, self-efficacy gains, and advocacy engagement than participants in a control group.

Methods

Promotoras and participants completed surveys that evaluated knowledge gains, behavior changes, self-efficacy, and advocacy engagement related to lowering exposures to toxins to reduce risks of breast cancer and reproductive harm. After completing the training, each of the 21 Promotoras recruited and educated 8-10 participants, who included family, friends, and/or community members. Once educated, participants were randomized into two groups: a control group and an intervention group. The intervention group received text messages to reinforce behavior change and provide ongoing education. The control group did not receive text messages or other reinforcements. Both Promotoras and participants were incentivized with gift cards.

Results

A total of 239 participants were educated by the Promotoras through 42 group sessions. Although results were not statistically significant, the 129 participants in the intervention group demonstrated more promising overall behavior changes related to reducing exposure to toxins as compared to the 114 participants in the control group. Participants in both groups demonstrated an increase in knowledge of 67% to 84% in filtered tap water being safer than bottled water, and 65% to 88% in knowledge of exposure to plastic and cleaning products with fragrances linked to problems during pregnancy.

The 24 Promotoras recruited were of primarily Mexican origin (54%, 13), with 42% (10) having completed high school and 42% (10) in the lowest income bracket of $0 to $10,350. Once trained, 13 Promotoras' knowledge of toxins related to household toxins increased by 185%. There were 10 Promotoras whose confidence in their ability to reduce exposures increased by 249%. In terms of educating, confidence of seven Promotoras increased by 63%. Advocacy engagement grew exponentially, with petition signing increasing from 8.3% to 63% (2-15), and the percentage of Promotoras who had or intended to contact elected officials rose from 8.3% to 54% (2-13).

Conclusions

The Promotoras and participants deepened their knowledge, adopted healthier and safer behaviors in reducing exposures to toxins, and strengthened their commitment to advocacy. The foundation of this model is based on peer support combined with an evidence-based curriculum following best practices. The findings highlight the effectiveness of culturally and linguistically tailored health education led by trusted community members.

## Introduction

Women in under-resourced communities face heightened exposure to environmental toxins associated with an increased risk of breast cancer and reproductive harm. In Los Angeles, low-income Hispanic communities disproportionately face high levels of pollution. These regions, often referred to as “toxic hot spots,” are plagued by poor air quality due to urban oil drilling and industrial emissions, further jeopardizing the health of these communities. Low to moderate-income Latinas of reproductive age (15-40 years old) are particularly vulnerable to these toxins. These women face significant challenges, including limited financial resources and restricted access to health-promoting environments. Notably, Latinas make up the largest proportion of women in Los Angeles County at 46%, and approximately 14.1% of Latinas aged 18 to 64 are uninsured [1]. To address these health disparities, Promotoras, lay health educators, were trained to focus on the impact of environmental toxins on breast cancer and reproductive harm. The Iris Cantor-UCLA Women’s Health Education & Research Center (WHERC) and Worksite Wellness LA (WWLA) collaborated to develop a risk-reduction and environmental advocacy training program. 

The geographic focus for this study was South Los Angeles, specifically within Los Angeles Service Planning Area (SPA) 6, where the impact of these environmental and socioeconomic factors is significant. This area has the second-highest mortality rate for breast cancer in Los Angeles County, according to the 2017 Key Indicators of Health report from the Los Angeles County Department of Public Health [1]. The American Cancer Society reports that breast cancer is the most commonly diagnosed cancer among Hispanic women and remains the leading cause of cancer-related death among Latinas in the United States [2]. Despite these alarming statistics, access to health promotion and disease prevention services remains limited. This is due to barriers including lack of insurance, inability to pay for services, and cultural and language obstacles. 

Effects of pollution experienced by pregnant women include heightened risks of adverse outcomes such as preeclampsia, intrauterine growth restriction, and spontaneous abortion [3]. Exposure to air pollutants such as ozone and fine particulate matter has also been linked to low birth weight and stillbirth [4]. In addition, heavy metals found in breast milk pose serious health risks to newborns [5]. Exposure to endocrine-disrupting compounds (EDCs), including phthalates, parabens, and polyfluoroalkyl substances (PFAS), negatively affects ovarian function, fertility, and the timing of menarche and menopause [6]. These disruptions have far-reaching implications for other aspects of women’s health, including mental health disorders, cardiovascular disease, and other cancers [7]. Short-lived exposures to EDCs during conception, pregnancy, or early childhood can result in long-term health consequences for both women and future generations [8].

Links between breast cancer, reproductive harm, and environmental toxins 

Environmental exposures are recognized by the National Institute of Environmental Health Sciences as contributing factors to breast cancer risk [9]. Research has identified several environmental toxins, including polycyclic aromatic hydrocarbons (PAHs), EDCs such as phthalates and parabens, and persistent pollutants like dioxins and PFAS, as potential carcinogens associated with increased breast cancer risk [10-13]. For example, Shen et al. found that women with high levels of PAHs in their blood had a threefold increase in breast cancer risk [12].

Occupational and residential exposures further compound these risks. Cadmium, a metalloestrogen, has been shown to activate estrogen receptors and is associated with a 50% to 130% increased breast cancer risk among women with occupational exposure [14]. Dioxins, often released through industrial waste and incineration, have been linked to increased breast cancer risks for women residing within ten kilometers of a waste incineration facility, with elevated risks at closer distances [15].

Harmful exposures can also come from household products. Many conventional cleaning agents contain EDCs, which accumulate in the body over time and can be transferred to nursing children through breast milk [16]. Volatile organic compounds (VOCs), commonly found in surface cleaners and air fresheners, are also linked to hormone disruption and reproductive harm. The U.S. Environmental Protection Agency (EPA) reports that indoor air pollution levels are often higher than outdoors, primarily due to VOCs [17].

EDC exposures during critical windows, such as conception, pregnancy, and early childhood, can have long-lasting effects across generations [18]. Lead, the first widely recognized EDC, is known to impair central nervous system development, leading to cognitive delays and behavioral issues in children [19]. Children from low-income households are disproportionately affected by lead exposure, compounding educational disparities and perpetuating cycles of poverty.

Environmental injustice linked to racial and health disparities

Marginalized communities, particularly low-income neighborhoods, communities of color, and Indigenous populations, bear a disproportionate burden of environmental pollution. Due to systemic inequities in zoning, land use planning, and environmental enforcement, these communities are more likely to be near waste sites such as incinerators, oil drilling sites, and landfills [20]. The result is elevated exposure to harmful substances in the air, water, and soil, contributing to chronic diseases, reproductive harm, and respiratory issues.

In Los Angeles, neighborhoods such as South LA are home to the largest urban oil fields in the U.S., further illustrating this pattern. Residents, who are predominantly Black and Latino, are chronically exposed to industrial toxins and face elevated risks of cancer, asthma, and adverse birth outcomes. Nearby waste sites release hazardous materials such as asbestos, lead, and mercury into the surrounding environment, contaminating air and groundwater [21]. A review of 50 publications from 1980 to 1988 reported increased risks of low birth weight and birth defects for individuals living within one mile of landfills, with higher risks associated with proximity to hazardous waste sites [22].

These environmental burdens are often compounded by social stressors such as overcrowded housing, food insecurity, and limited access to healthcare. Addressing these interconnected inequities requires more than technical interventions-it demands community-driven advocacy, equitable policy reform, and long-term investment in the resilience of historically neglected neighborhoods. Addressing these health disparities can effectively leverage the lived expertise of Promotoras, also known as community health workers. These Promotoras are well-positioned to mobilize their communities to advocate for systems change. 

Community-based interventions

Training Latinas as Promotoras is an evidence-based strategy for addressing health disparities in historically underserved communities [23]. As trusted and culturally connected members of their communities, Promotoras have built meaningful relationships, allowing them to effectively share health information, model behavior change, and empower others through education, advocacy, mentorship, and outreach. This model is compelling in communities where language barriers, immigration concerns, and historical mistrust of medical institutions often deter individuals from accessing conventional health systems.

Research shows that Promotora-led interventions improve health knowledge and behavior and can lead to long-term improvements in confidence, social support, and collective action. In one qualitative study, Latinas reported feeling more empowered to make health-related decisions for themselves and their families after participating in a Promotora-led lifestyle program [24]. These interpersonal dynamics make Promotoras uniquely equipped to advance environmental health literacy in ways that are culturally resonant and accessible.

Community-Based Participatory Research (CBPR) aligns naturally with the Promotora model by ensuring that interventions are community-driven, culturally appropriate, and grounded in lived experience [25]. CBPR supports the design of effective interventions and strengthens environmental justice efforts by centering community voices and redistributing power. Mobile text messaging was introduced to reinforce participant engagement beyond the Promotora-led educational sessions to enhance Promotora-led outreach. A 2017 American Journal of Preventive Medicine meta-analysis found that health-focused text message interventions effectively promote sustained behavior change and can influence participants past the end of an intervention [26]. These findings reinforce the value of integrating low-cost, scalable digital strategies into community-based health promotion. Applying this model to the Promotora program provided an opportunity to evaluate its applicability and effectiveness in yielding higher success rates for the intervention group. 

## Materials and methods

Promotora training program

Applying the Promotora model to reduce community health risks from environmental toxin exposures, the Iris Cantor-UCLA Women’s Health Education & Research Center (WHERC) and Worksite Wellness LA (WWLA) developed a health education program titled “Promotora Training: Reducing Environmental Toxins on Cancer and Reproductive Health.” The program consisted of training women as Promotoras to educate family, friends, and community members about risk reduction strategies and how to engage in advocacy. There were a total of 24 Promotoras recruited. A total of 21 completed the training and subsequently conducted educational sessions in the community.

The Promotora training program was held via Zoom in February 2024 and was led by a trained facilitator from WWLA. Each session included both didactic instruction and interactive components. The sessions were structured as follows: Session 1: Role and Expectations of a Promotora, Session 2: Toxins, Reproductive Health, & Breast Cancer, Session 3: Toxins and Health Equity, and Session 4: Adopting the Role of a Health Educator

The primary objective of this California Breast Cancer Research Program (CBCRP) funded study was to promote environmental justice and related risk-reduction behaviors by mobilizing and educating Latinas who reside in South and Central Los Angeles about the impact of environmental toxins on breast cancer and reproductive health. The initiative aimed to educate Latinas about environmental toxins and empower them to adopt personal practices to reduce breast cancer risks and reproductive harm, protect their health, and equip them with advocacy skills to catalyze broader environmental justice advocacy efforts within the community. A website was created where training materials were posted, fostering a learning community and providing access to the entire training curriculum and additional educational resources on environmental toxins, breast cancer, reproductive harm, and advocacy engagement.

All Promotoras were asked to complete both demographic and evaluation surveys at two points: once before the first session and once following the final session. Surveys assessed knowledge, behavior, self-efficacy, and community advocacy engagement. Upon completing the training, each Promotora was responsible for leading two educational sessions for family, friends, or community members within the following six weeks. 

The first session focused on exposures from the built environment and other exposures encountered in daily living. The second session emphasized advocacy and community action. The Promotoras focused on supporting participants in adopting both personal and collective action to reduce exposure to environmental toxins and minimize the risks of breast cancer and reproductive harm. A total of 24 Latinas were enrolled in the Promotora training program. Once trained, Promotoras were responsible for recruiting eight to ten community members from their social networks to participate in two educational sessions. Participants were randomized to participate in the text messaging intervention or the control group. 

With respect to the Promotoras, the impact of the educational and training program was evaluated in four dimensions. First, Promotoras' knowledge levels about modifiable risk factors associated with breast cancer and reproductive harm were measured in terms of their confidence in educating others. Second, we measured adjusted behaviors reducing exposure to toxins. Third, the Promotoras' level of self-efficacy in educating peers was measured. Fourth, we evaluated levels of advocacy engagement amongst the Promotoras in environmental justice campaigns. 

Promotora recruitment 

WWLA, a nonprofit organization that serves predominantly low-income, uninsured Latinx populations in South and Central Los Angeles, conducted all recruitment for this program by accessing their vast network of Promotoras and strong community presence to identify candidates for the training. Most Promotoras were between the ages of 25 and 54, with 54% identifying as of Mexican origin. Approximately 42% of participants reported having completed high school or a GED, and 42% identified as belonging to the lowest income bracket. Spanish was the primary language spoken in 88% of households. Most Promotoras reported being the primary caregiver in their home and being responsible for decisions related to food, cleaning products, and household health practices. Eligibility criteria included identifying as Latina, being of reproductive age (15-40), and residing in South or Central Los Angeles areas designated as environmental “hot spots” due to high pollution burden and socioeconomic vulnerability. 

Participant recruitment 

The Promotoras recruited participants from their circle of friends, neighbors, family members, and their networks of parents and other community and civic groups. The Promotoras scheduled their education sessions according to their schedules. A total of 239 Latina participants attended the educational sessions. Due to privacy concerns, specific demographic/identifying questions regarding income, education, and age were not asked in participant surveys. 

Text messaging intervention

A supplemental text messaging randomized controlled trial was implemented after the initial training to test if the education provided by Promotoras enhanced and reinforced behavior change. Through cluster randomization by Promotoras, community participants were placed into control or intervention groups. The messages provided nudges to reinforce the education and provide complementary facts and advice gained in the sessions conducted by Promotoras. 

Of the 21 Promotoras who completed the training and conducted educational sessions, 11 had their participants assigned to the intervention group, and 10 had their participants assigned to the control group. However, because each of the Promotoras conducted education sessions for 8-10 participants, their total number of participants was not the same. Therefore, the number of control and intervention group participants was not equal. 

The intervention group received 12 text messages over six weeks. Messages were delivered in Spanish and included actionable tips for reducing exposure to environmental toxins, reminders about risk-reduction behaviors discussed in educational sessions, links to culturally relevant resources, and digital stories showcasing community members who had made successful health-related changes. The control group did not receive any text messages. The goal was to determine whether the additional educational messages and “nudges” delivered via text to the intervention group could lead to more significant gains in knowledge and behavior change than the control group. 

Data collection

Data were collected using pre- and post-surveys administered to Promotoras and community participants. The surveys included multiple-choice and Likert-scale questions and were designed to assess four key domains: environmental health knowledge, self-reported behavior change, self-efficacy, and engagement in advocacy activities. Surveys were developed in Spanish to ensure linguistic accessibility for all participants. Promotoras completed their surveys at three different time points: before the first training session, immediately following the fourth session, and six weeks after completing their last community education session. Similarly, community participants educated by the Promotoras completed surveys at the beginning and end of their first session, and six weeks later during a follow-up meeting. All surveys for Promotoras and participants were intended to be administered via a REDCap link accessible via phone or computer. However, due to digital access limitations, unfamiliarity with completing online surveys, and preferences for printed surveys, participants completed surveys on paper. The data was later tabulated in a digital format. 

Completed surveys were tracked by Promotoras, who then notified WWLA staff. Although completing the survey entitled Promotoras to receive a gift card, the trust built between the Promotoras and program facilitators was strong enough to believe they were honest about the number of completed surveys reported. WWLA securely stored paper surveys throughout the duration of the study. All online data were securely stored in a password-protected database managed by the UCLA Department of Medicine Statistical Core (UCLA DOMStat). Only the UCLA DOMStat project staff involved in the analysis and the WWLA project staff had access to raw data. 

Statistical methods

Due to limitations in linking participant surveys pre-to-post test, data at all time points were summarized independently. Evaluations of change emphasized differences in group-level statistics. Analyses were performed using the 2013 Statistical Analysis System SAS version 9.4 (SAS Institute Inc., Cary, NC). All tests used a two-sided alpha of 0.05. For bivariate tests of association between the randomization arm (control vs. intervention) and survey items, or time (pre vs. post) and survey items, we used chi-square tests or Fisher's exact tests when appropriate. To test for differences in the effect of timing (pre vs. post) on survey items between randomization arm (control vs. intervention), we utilized logistic regression models (binary and multinomial logistic regression models, depending on the number of response categories of a given survey item) with an interaction term between the timing and randomization arm.

## Results

Data analysis

Table 1 demonstrates examples of actions discussed in the educational training and sent as text messaging nudges to the intervention participant group. Although only a handful of p-values demonstrated significance, action participation due to the nudges sent to the intervention group was greater in the intervention group overall as compared to the control group. 

**Table 1:** Participant Outcomes - Behavior Change

Action	Overall (n=212)	Control (n = 98)	Intervention (n =114)	p-value	Chi-square value
Storing food in glass or ceramic containers instead of plastic.	139 (65.57%)	59 (60.20%)	80 (70.18%)	0.1277	2.3207
Cleaning my home with non-toxic ingredients like vinegar and baking soda.	134 (63.21%)	56 (57.14%)	78 (68.42%)	0.0896	2.8824
Using a damp cloth or mop to clean instead of a dry cloth or sweeping.	112 (52.83%)	47 (47.96%)	65 (57.02%)	0.1877	1.7352
Other (i.e., washing fruits and vegetables with a mixture of vinegar and water, selecting to buy organic foods when possible)	15 (7.08%)	3 (3.06%)	12 (10.53%)	0.0346	4.4666

Table 2 demonstrates similar results to those previously stated. Despite only a few p-values demonstrating significance, greater response rates in this question explain the possible realization among participants of knowing how to limit toxin exposures following attending a Promotora-hosted education session but also recognized barriers that they encountered when trying to make those changes. 

**Table 2:** What Gets in the Way of Making Changes That Would Reduce Your Exposure to Toxins? (Check All That Apply)

	Overall (n=212)	Control (n=98)	Intervention (n=114)	p-value	Chi-square value
Resources – nontoxic products are too expensive.	44.34%	53.06%	36.84%	0.0178	5.6171
Access – it’s difficult to find nontoxic products in my neighborhood.	33.49%	32.65%	34.21%	0.8107	0.0573
Habits – it’s difficult to get used to new habits.	56.13%	46.94%	64.04%	0.0124	6.2553
Family – the people in my family don’t want to make changes.	18.40%	16.33%	20.18%	0.4708	0.5200
Skepticism – I’m not sure how much of a difference it will really make.	4.25%	3.06%	5.26%	0.5099	0.6284
Busy – I don’t have time to focus on making changes.	6.13%	5.10%	7.02%	0.5622	0.336
Preferences – I don’t want to stop using the products I already use.	6.13%	4.08%	7.89%	0.2486	1.3311
Confusion – I’m unclear which options are the safest.	3.77%	3.06%	4.39%	0.7277	0.2547
Other ____________________	4.25%	6.12%	2.63%	0.3078	1.5798

Behavioral and lifestyle recommendations were sent as “nudge” text messages to participants in the intervention group. Overall, greater participation and engagement from the intervention group demonstrate the benefits of these “nudges” in promoting participants to adopt behaviors reducing their toxin exposure, thus lowering their risks of breast cancer and reproductive harm. Motivation to implement toxin-reducing behaviors was higher in the intervention group in eight out of nine categories, 89%, compared to the control group, one out of nine categories, 11%. 

Intervention group participants also reported greater confidence in motivating others to reduce their exposure to toxins, most notably in the “completely confident” and “slightly confident” categories. The text messages emphasized using affordable and easily accessible products like vinegar and baking soda to make nontoxic cleaning solutions. They further recognized that health or beauty products are expensive and not easily accessible in their neighborhoods, and they were given information on making their own non-toxic alternatives. 

Promotora outcomes

Knowledge Gains

Post-training surveys revealed significant improvements in knowledge across several key areas of environmental health. For example, recognition of PFAS as harmful chemicals found in fast fashion increased from 29% to 83%. Awareness of toxins in furniture increased from 21% to 54%, and knowledge of harmful ingredients in cleaning products improved from 33% to 58%. These gains suggest the training effectively raised awareness of environmental risks and their sources of toxins.

Behavior Change

Promotoras reported meaningful changes in behavior aimed at reducing environmental toxin exposure. For instance, the percentage of participants who actively avoided products with harmful chemicals increased from 46% to 67%. Self-reported use of non-toxic alternatives in cleaning and food storage increased by 13% to 21% across different categories. Additionally, 38% of Promotoras indicated they had shared toxin-reducing practices with others beyond the attendees at their educational sessions.

Self-Efficacy

The trained Promotoras’ confidence in their ability to reduce personal exposure to environmental toxins improved, as did confidence in educating others. On average, participants rated their self-efficacy 29% higher after the training. Thus, Promotoras in this program expressed a stronger sense of their ability to adopt healthier behaviors and help others do the same.

Advocacy Engagement

Civic engagement in advocacy campaigns increased significantly. Promotoras who reported signing petitions grew from 8.3% at baseline to 63% post-training. Those who contacted elected officials jumped from 8.3% to 54%. These results suggest that peer-to-peer education sparks broader community advocacy.

Participant outcomes

Knowledge Gains 

Overall, just in the first session, participants in both the intervention and control groups demonstrated an increase in knowledge of 67% to 84% in filtered tap water being safer than bottled water and 65% to 88% in knowledge of exposure to plastic and cleaning products with fragrances causing problems during pregnancy. 

Self-Efficacy

**Figure 1:**
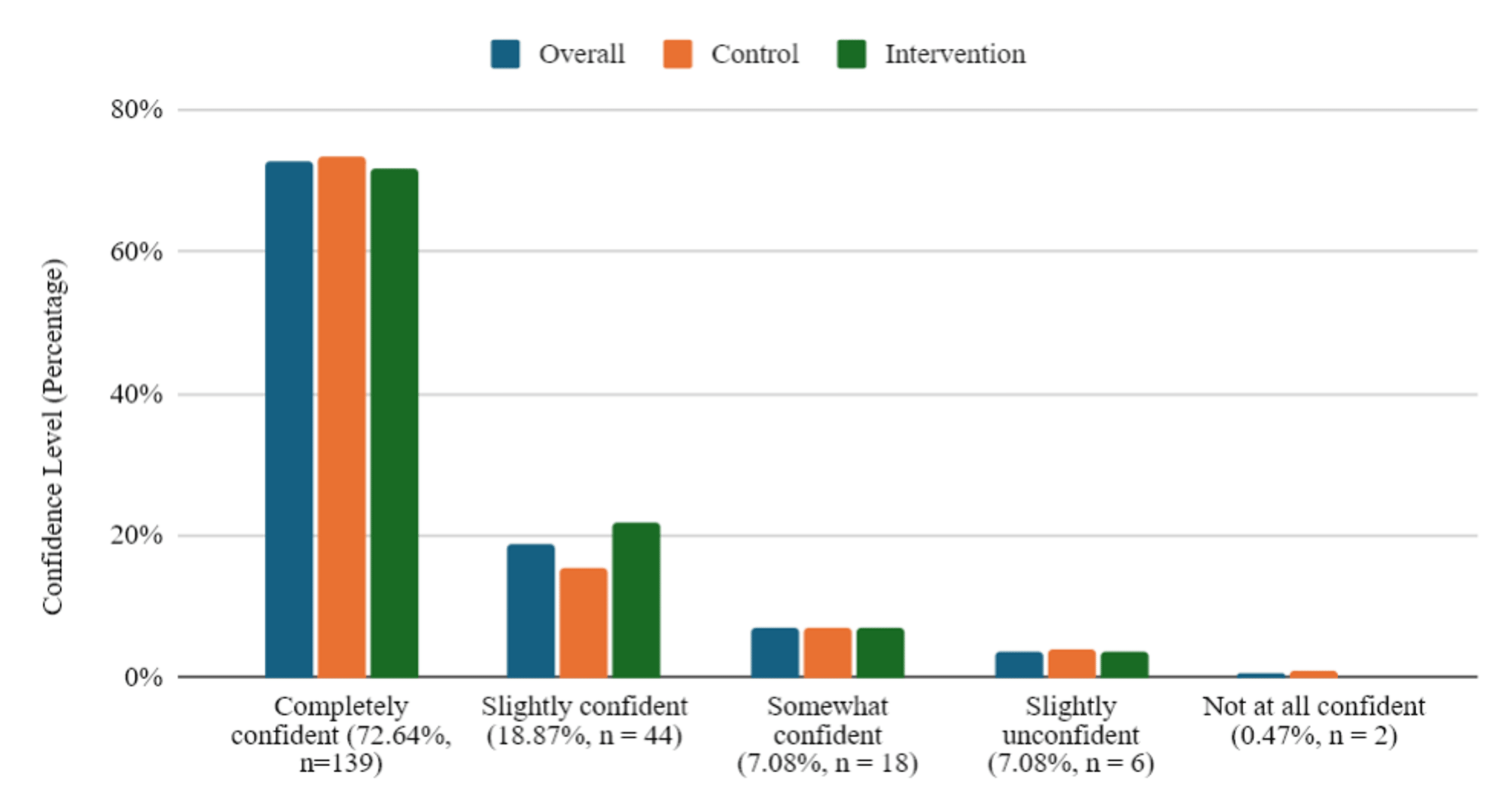
Participants' Outcomes: Confidence Levels in Their Ability to Reduce Exposures to Toxins (n = 209)

As seen in Figure 1, participants gained confidence in reducing toxin exposure 6 weeks after the first session (139 participants (66%) selected completely confident, 45 selected slightly confident (21%), and 20 selected somewhat confident (9.4%), amounting to a total of 96.4% participants demonstrating some initial confidence in their ability in reducing toxin exposures. 

Advocacy Engagement

Advocacy results were inconsistent in both the control and intervention groups, depending on the level of involvement of each advocacy behavior. Intervention group participants shared greater participation (14%) in attending a community advocacy event about environmental justice, while control group participants had only 10% participation in community advocacy events. Overall, control and intervention group participants demonstrated similar levels of advocacy participation, with some activity participation being greater in the control group. As noted in Table 3, barriers to advocacy engagement in both intervention and control groups included not having enough time, not being aware of ways to get involved, not understanding what is being advocated enough to become involved, and their beliefs about whether their involvement in advocacy would make a difference.

**Table 3:** Participant Outcomes: Advocacy Engagement Barriers

	Overall (n=212)	Control (n=98)	Intervention (n=114)	p-value	Chi-square value
I am not aware of specific ways to get involved in advocacy activities.	35.38	37.76	33.33	0.5020	0.4506
I don’t understand the issues being advocated for well enough.	8.96	4.08	13.16	0.0211	5.3206
I am skeptical that it will make a difference.	10.85	15.31	7.02	0.0530	3.7432
I don’t have enough time.	40.57	39.8	41.23	0.8323	0.0448
I feel overwhelmed by which environmental issues are most important.	9.91	6.12	13.16	0.0873	2.9228
I am afraid of challenging authority.	6.13	5.10	7.02	0.5622	0.3360
No response	4.72	3.06	6.14	0.3464	1.1116

## Discussion

Key findings

Promotoras

The results of this evaluation validate the effectiveness of the Promotora model in educating and engaging marginalized populations in developing and delivering health education programs. A literature search did not identify any previous studies or outcome data of training lay community health advocates in building environmental health knowledge, promoting behavior change, and empowering participants to engage in community education and advocacy. However, evidence of positive outcomes from Promotoras in other settings addressing health issues, such as tobacco control and promoting healthy lifestyles, demonstrates the impact of Promotoras as educators and influencers [27,28].

Our study observed gains across all measured domains: knowledge, behavior, confidence, and civic participation. Post-training data revealed substantial increases in attaining correct knowledge of environmental toxins, including awareness of per- and polyfluoroalkyl substances (PFASs) in fast fashion, furniture, and cleaning products. Confidence in reducing exposure and educating others also rose by 29-46%, and behavior changes, such as avoiding harmful cleaning products or using gloves with bleach, increased by 13-21%.

The most dramatic improvement occurred in Promotora advocacy. Before the training, only 8.3% of Promotoras had ever signed a petition or contacted an elected official. After the training, these numbers jumped to 63% and 54%, respectively. These outcomes support the value of Promotoras in delivering culturally and linguistically tailored community-based education that influences individual health behaviors and seeds grassroots leadership and civic engagement in communities historically burdened by environmental injustice. 

Participants

Although 239 participants were educated, 243 pre-test surveys were completed, four more than the number of participants. It is possible that there were duplicate surveys completed by participants due to confusion regarding the surveys or misremembering whether they had completed them or not. It could also be attributed to human error due to the fact that the paper surveys were manually input into the REDCap system. 

For the six-week follow-up survey, 212 surveys were completed, 27 fewer than the 239 participants. This may be due to too much lag time between the education sessions and the follow-up survey, where participants were not interested in completing another survey despite the incentive of possibly winning the raffle gift card. 

The text messaging intervention showed potential as a low-cost reinforcement tool, offering continued “nudges” to encourage adoption of healthy behaviors among program participants [26]. Although this component warrants further analysis, early comparisons between intervention and control participants suggest that combining digital text messaging with peer-led education holds promising value. Our results mirror other studies where interventions that included elements in addition to text messaging were not necessarily more successful than interventions solely using text messaging.

One factor that may have affected lower selection rates for the final response survey was survey fatigue. This may be due to having too many questions and too many options to select from within the final response survey. Barriers to behavior change mentioned on the survey included a lack of knowledge within the broader community, insufficient motivation, and entrenched habits. Additionally, intervention participants shared their obstacles to advocacy engagement. Some noted experience limited to no barriers, citing participation with other local environmental justice advocacy organizations, while others identified challenges to getting involved in advocacy engagement, including time, awareness, and confidence in taking action. 

Limitations

Despite the program’s success, several limitations were identified. Operational and logistical challenges occurred frequently, particularly regarding maintaining timely follow-up and ensuring consistent data collection. Due to varying levels of digital literacy across participants, surveys were distributed and completed manually, requiring the research team to scan and enter all responses into REDCap. This effort-intensive process increased the risk of transcription errors and added a considerable unanticipated workload to the study team.

The Promotoras’ profile was expanded beyond the original inclusion age criteria of 18-40 and geographic region to reach 24 participants. This was due to WWLA’s vast network of Promotoras outside of South LA who were beyond reproductive age and were eager to participate. Furthermore, Promotoras experienced challenges maintaining contact with their community participants, scheduling educational sessions, and ensuring surveys were completed appropriately. As a result, the research staff remained highly engaged beyond the expectations outlined in the study protocol and budget, offering additional support and creative scheduling strategies to ensure data integrity.

With regard to the text messaging intervention, given that the control group had 114 participants, 15 fewer than the intervention group of 129, the differences in numbers were minimal. Nevertheless, randomly selecting control and intervention participants rather than equally dividing the Promotoras to have their participants assigned to the intervention or control groups would have been a more evidence-based approach. Not having equal participants in the control and intervention groups was a limitation, yet it did not necessarily impact the outcome results. 

We were unable to perform a McNemar's test, which would have shown the individual changes in participants throughout the educational sessions and intervention [29]. There were obstacles in linking participants consenting to have their pre-, post-, and follow-up surveys linked due to privacy and confidentiality concerns. Despite the participants' trust in the Promotoras who conducted the sessions, participants displayed a hesitancy in sharing more personal information with the Promotoras. 

## Conclusions

This study’s Promotora model fostered trust among community members, leveraged peer support, and integrated cultural relevance. This study yielded promising data that can be further studied and confirmed in a cluster trial with other variables considered, like measures of exposure, as well as incorporating longitudinal data. Community-driven education programs are powerful tools for advancing health equity and environmental justice. Future initiatives that provide structured pathways for sustained advocacy and policy engagement have the most potential to be impactful. Cross-sector partnerships with policy organizations and working with housing, environmental, and community development agencies may also strengthen upstream strategies to reduce environmental toxin exposure and build healthier communities. 

Scaling and adapting this model in other under-resourced communities across the U.S. holds great promise, particularly when paired with a digital messaging component and ongoing support networks. Promotoras have been shown to be powerful facilitators in reducing risks for overall breast cancer and reproductive harm. They are essential partners in achieving environmental justice and health equity. 

## Appendices

**Table 4:** Study Design Overview

	IRB Approved Protocol	Protocol Modifications
Promotora Age Range	18-40	Age expanded to 54 to meet recruitment goals (20 Promotoras)
Geography	South Los Angeles	South and Central Los Angeles
Survey Method	Digital (Qualtrics)	Switched to paper-based survey due to digital literacy barriers
Participant Follow-up	3-month post survey	3-month follow-up not conducted
SMS Text Message Intervention Duration	12 weeks	Shortened to 6 weeks
Statistical Analysis	McNemar’s Test, Diff-in-Diff	McNemar’s Test was not conducted; only descriptive statistics reported | 2013 statistical analysis SAS version 9.4. | All tests used a 2-sided alpha of 0.05. | For bivariate test, Chi-square test or Fisher’s exact test when appropriate | To test for differences in the effect of timing, utilized logistic regression models
Digital Platform	Program Website	No sustained engagement post program

**Table 5:** Promotora Pre-Test Questions

Question:	Response Choices:
Demographics:	
What is your age?	18-24 | 25-34 | 45-54 | 55-64 | 65 and over
What is your gender?	Female | Non-binary | Prefer not to say
What is your marital status?	Single | Married | Divorced | Separated | Widowed | Prefer not to say
What is your ethnic background?	Mexican | Salvadoran | Guatemalan | Other Latino | I don't know | Prefer not to answer
What is your zip code?	(Open response)
What is your highest level of education completed?	Less than high school | High school graduate | Some college | College graduate
What is your current employment status?	Part-time work | Full-time work | Self-employed | Homemaker | Seeking employment | Unemployed | Retired | Other
Which of the following describes your individual income last year?	$0 - $10,350 | $10,351 - $26,500 | $26,501 - $44,150 | $44,151 - $70,650 | Prefer not to answer
Knowledge-Based Questions:	
At which stage can environmental exposure to toxins be harmful to the developing fetus?	Neonatal Stage | Menopause Stage | Embryonic Stage | Lactation Stage
Which of the following systems in the human body can be affected by microplastics? (check all that apply)	Reproductive system | Nervous system | Endocrine system | Cardiovascular system | Respiratory system
There are per-and polyfluoroalkyl substances (PFAS) chemicals in the following: (check all that apply)	Fast fashion | Ceramic dishes | Furniture | Glass containers | Cleaning products | All of the above
In simple terms, the precautionary principle is:	To prioritize economic interests over environmental concerns | To delay decision-making | To be better safe than sorry | To make decisions quickly
Endocrine disruptors are:	Harmful chemicals that affect the endocrine system | Compounds that mimic or interfere with the body's endocrine system | Substances that have no impact on hormonal systems | Vitamins are essential for hormonal balance | Elements crucial for hormonal balance | Compounds that enhance the endocrine system
Self-Assessment of Confidence:	
How confident do you feel in your ability to reduce your exposure to toxins? (linear scale)	1 (Not at all Confident) | 2 | 3 | 4 | 5 (Very Confident)
How confident do you feel about motivating others to take steps to lower their exposure to environmental toxins? (linear scale)	1 (Not at all Confident) | 2 | 3 | 4 | 5 (Very Confident)
How confident do you feel about motivating others who express barriers (financial difficulties, personal difficulties, etc). In making a change?	1 (Not at all Confident) | 2 | 3 | 4 | 5 (Very Confident)
Fill in the blank: In the last 6 months, I have made __ changes to my lifestyle choices regarding environmental toxins. (This question is a work in progress. Any suggestions/recommendations for improving this question would be greatly appreciated.	1-2 | 3-4 | 4-5 | 6+
Background to Advocacy:	
In the last 6 months, which of the following advocacy activities have you engaged in? Check all that apply	Signed a petition | Attended a community advocacy meeting | Attended a community advocacy event (protest, march, etc) | Sharing information on social media about environmental hazards | Donated to an organization with a mission of cleaning up the environment | Participated in a community clean-up event (i.e., beach clean-up) | Met with an elected official | Contacted (phone/email) an elected official | Other
What motivates you to become an advocate for your community's health? Please share your reasons. Check all that apply	Personal or family experiences with health issues | Desire to empower and educate the community |Belief in the importance of a healthier environment | Recognition of the impact of environmental factors on health | Commitment to social justice and community well-being | Faith-based reasons | Other
What are your challenges in advocating for your community's health? Check all that apply. (check all that apply)	Lack of specific ways to get involved in advocacy | Not understanding the issues being advocated for | Skepticism about the impact of community advocacy | Time constraints | Other
Learning about environmental toxins and their impact on breast cancer has…	Not concern me at all | Slightly concerned me | Somewhat concerned me | Moderately concerned me | Extremely concerned me
Learning about environmental toxins and their impact on reproductive health has…	Not concern me at all | Slightly concerned me | Somewhat concerned me | Moderately concerned me | Extremely concerned me

**Table 6:** Promotora Post-Test Questions

True or False:	
Most chemicals in products available in stores have been tested for safety.	True | False
It is better to dust with a wet cloth or mop than to sweep or use a dry cloth.	True | False
Insect traps are better than sprays.	True | False
House paint, soil, and dust never contain lead.	True | False
Canned foods may contain plastic.	True | False
Everyday Living Activities:	
I use personal care products that don't have toxins.	Always | Often | Sometimes | Rarely | Never | I don’t know if the products I use have toxins
I clean my home with cleaning products that don't have toxins, like baking soda and vinegar.	Always | Often | Sometimes | Rarely | Never
When storing food, I use glass or ceramic containers instead of plastic containers.	Always | Often | Sometimes | Rarely | Never
I take my shoes off at the door so that toxins from outside don't come inside the home.	Always | Often | Sometimes | Rarely | Never
I eat frozen or fresh fruits and vegetables instead of canned foods.	Always | Often | Sometimes | Rarely | Never
Knowledge of Promotora Role:	
The roles and expectations of a promotora are:	Community outreach | Community education | Community advocacy | Providing resources | All of the above
Community Advocacy:	
I feel comfortable advocating for healthier communities in my role as a promotora.	Not Comfortable at All | Slightly Comfortable | Moderately Comfortable | Very Comfortable | Extremely Comfortable
I have participated in the following advocacy activities (check all that apply).	Sign a petition | Attend a community advocacy meeting | Attend a community advocacy event (ex. protest, march) | Share information on social media about environmental hazards | Donate to an organization with a mission of cleaning the environment | Participate in a community cleanup event (ex. Beach cleanup) | Meet with an elected official | Contact an elected official through phone/email | I do not want to participate in an advocacy activity
I believe promotoras can significantly contribute to raising awareness about preconception health in their communities.	Strongly Disagree | Disagree | Neutral | Agree | Strongly Agree
Self-Assessment:	
On a scale of 1 to 5, with 1 being the lowest and 5 being the highest, how confident do you feel in advocating for healthier communities?	1 (Lowest Confidence) | 2 | 3 | 4 | 5 (Highest Confidence)
On a scale of 1 to 5, with 1 being the lowest and 5 being the highest, how confident do you feel about educating others on the link between environmental toxins and breast cancer and reproductive health?	1 (Lowest Confidence) | 2 | 3 | 4 | 5 (Highest Confidence)

**Table 7:** Promotora 6-week Follow-up Survey Questions

Question:	Response Choices:
How do you rate your advocacy skills for reducing toxin exposure in your community after completing the 2 community education sessions?	I saw little to no improvement in my advocacy skills | I saw some improvement in my advocacy skills | I saw moderate improvement in my advocacy skills | I saw a significant improvement in my advocacy skills | I saw substantial improvement in my advocacy skills
Roses: What successes did you find in providing classes to community members? (check all that apply)	Inspired community members to make changes to reduce exposure to toxins | Established a relationship of trust with community members | Gained confidence in providing health education to my community | Satisfaction from community members who appreciated learning about toxins | Other
Thorns: What challenges did you face with the program participants? (check all that apply)	Recruiting community members to attend sessions | Being asked questions, I didn’t know how to respond to | Community members' skepticism about the issues | Difficulty finding a time and date that worked for all program participants | Addressing conflicting information within the group | Other
How confident are you in your health education skills used during the two community sessions? On a scale of 1 to 5:	1 (Not at all Confident) | 2 | 3 | 4 | 5 (Very Confident)
How would you rate the support provided by the training staff in preparing you to provide the community classes? On a scale of 1 to 5:	1 (Not at all Supported) | 2 | 3 | 4 | 5 (Very Supported)
How helpful did you find the education materials provided for conducting the community education? On a scale of 1 to 5:	1 (Not at all helpful ) | 2 | 3 | 4 | 5 (Very helpful)
How confident are you in educating community members about reducing their exposure to toxins?	Not Confident | Somewhat Confident | Moderately Confident | Confident | Very Confident
Name, email, phone number (including area code)	Open Response

**Table 8:** Participant Pre- and Post- Test Questions

Question:	Response Choices:
Before the Program:	
Bottled water is safer than filtered tap water.	True or False
It’s better to dust with a wet cloth than a dry cloth.	True or False
Pesticides increase the risk of reproductive health issues.	True or False
Which of the following can contain lead?	Dust | Dirt | Peeling paint | All of the above
Exposure to which of the following can cause problems during pregnancy?	Plastic | Cleaning products with fragrances | Glass containers | Both Plastic and Cleaning products with fragrances
Consider whether the ingredients in the products I use daily have chemicals or not.	Often | Sometimes | Rarely | Never
Stay informed about environmental toxins that may impact me and my family.	Often | Sometimes | Rarely | Never
Store food in glass or ceramic containers instead of plastic containers.	Often | Sometimes | Rarely | Never
Avoid using products with synthetic fragrances.	Often | Sometimes | Rarely | Never
After the Program:	
Bottled water is safer than filtered tap water.	True or False
It’s better to dust with a wet cloth than a dry cloth.	True or False
Pesticides increase the risk of reproductive health issues.	True or False
Which of the following can contain lead?	Dust | Dirt | Peeling paint | All of the above
Exposure to which of the following can cause problems during pregnancy?	Plastic | Cleaning products with fragrances | Glass containers | Both a. and b.
Consider whether the ingredients in the products I use daily have chemicals or not.	Often | Sometimes | Rarely | Never
Stay informed about environmental toxins that may impact me and my family.	Often | Sometimes | Rarely | Never
Store food in glass or ceramic containers instead of plastic containers.	Often | Sometimes | Rarely | Never
Avoid using products with synthetic fragrances.	Often | Sometimes | Rarely | Never

**Table 9:** Participant Six-Week Follow-Up Survey Questions

Question	Response Choices
How confident do you feel in being able to reduce your exposure to toxins?	Completely confident | Slightly confident | Somewhat confident | Slightly unconfident | Not at all confident
In the last 6 weeks, which of the following changes have you made to reduce your exposure to toxins?	Drinking filtered tap water instead of bottled water. | Storing food in glass or ceramic containers instead of plastic. | Cleaning my home with non-toxic ingredients like vinegar and baking soda. | Reading the ingredient labels of personal care products to avoid toxins. | Reading the ingredient labels of cleaning products to avoid toxins. | Using insect traps instead of sprays or bug bombs. | Using a damp cloth or mop to clean instead of a dry cloth or sweeping. | Leaving my shoes at the door to avoid bringing toxins into my home. | Other________________________________________
What motivates you to make changes to reduce your exposure to toxins? (check all that apply)	I want to be as healthy as possible. | I have a family history of cancer and want to do what I can to avoid it. | I have a prior history of cancer and don’t want it to recur. | I am concerned for the health of my family/children. | I care about protecting the environment. | I care about protecting the health of people in my community. | Other ______________________
How confident do you feel in being able to motivate others (family, friends) to reduce their exposure to toxins?	Completely confident | Slightly confident | Somewhat confident | Slightly unconfident | Not at all confident
What gets in the way of making changes that would reduce your exposure to toxins? (Check all that apply)	Resources – nontoxic products are too expensive. | Access – it’s difficult to find nontoxic products in my neighborhood. | Habits – it’s difficult to get used to new habits. | Family – the people in my family don’t want to make changes. | Skepticism – I’m not sure how much of a difference it will really make. | Busy – I don’t have time to focus on making changes. | Preferences – I don’t want to stop using the products I already use. | Confusion – I’m unclear which options are the safest. | Other _________________________
In the last 6 weeks I have participated in the following activities related to the reduction of exposure to environmental toxins:	Participated in a community clean-up, such as picking up trash in my neighborhood or at the beach. | Signed a petition. | Attended a community advocacy meeting about environmental justice. | Attended a community advocacy event about environmental justice. | Other _________________ | I have not participated in an advocacy activity
What gets in the way of getting involved in advocacy activities that would help reduce exposure to environmental toxins? (Check all that apply)	I am not aware of specific ways to get involved in advocacy activities. | I don’t understand the issues being advocated for well enough. | I am skeptical that it will make a difference. | I don’t have enough time. | I feel overwhelmed by which environmental issues are most important. | I am afraid of challenging authority.
